# Population-Level Decline in BMI and Systolic Blood Pressure Following Mass HIV Treatment: Evidence from Rural KwaZulu-Natal

**DOI:** 10.1002/oby.21663

**Published:** 2016-12-07

**Authors:** Pascal Geldsetzer, Andrea B. Feigl, Frank Tanser, Dickman Gareta, Deenan Pillay, Till Barnighausen

**Affiliations:** 1 Department of Global Health & Population, Harvard T.H. Chan School of Public Health, Boston, Massachusetts, USA; 2 Department of Global Health and Social Medicine, Harvard Medical School, Boston, Massachusetts, USA; 3 Africa Health Research Institute, Mtubatuba, South Africa; 4 Institute of Epidemiology and Health Care, University College London, London, UK; 5 Institute of Public Health, Faculty of Medicine, Heidelberg University, Heidelberg, Germany

## Abstract

**Objective:**

Clinic-based studies have shown that patients with human immunodeficiency virus (HIV) gain weight after initiation of antiretroviral therapy (ART). This study aimed to determine whether the scale-up of ART was associated with a population-level increase in body mass index (BMI) and blood pressure (BP) in a community with high HIV and obesity prevalence.

**Methods:**

A household survey was conducted in rural KwaZulu-Natal before ART scale-up (in 2004) and when ART coverage had reached 25% (in 2010). Anthropometric data was linked with HIV surveillance data.

**Results:**

Mean BMI decreased in women from 29.9 to 29.1 kg/m^2^ (*P* = 0.002) and in men from 24.2 to 23.0 kg/m^2^ (*P* < 0.001). Similarly, overweight and obesity prevalence declined significantly in both sexes. Mean systolic BP decreased from 123.0 to 118.2 mm Hg (*P* < 0.001) among women and 128.4 to 123.2 mm Hg (*P* < 0.001) among men.

**Conclusions:**

Large-scale ART provision is likely to have caused a decline in BMI at the population level, because ART has improved the survival of those with substantial HIV-related weight loss. The ART scale-up may have created an unexpected opportunity to sustain population-level weight loss in communities with high HIV and obesity prevalence though targeted lifestyle and nutrition interventions.

## Introduction

Noncommunicable diseases (NCDs) are rapidly replacing infectious diseases as the leading causes of the disease burden in sub-Saharan Africa (SSA) (1,2). Yet the human immunodeficiency virus (HIV) epidemic continues to take a considerable toll in SSA, where approximately 26 million people were living with HIV in 2014 (3). More recently, there has been an increasing policy and research interest in the relationship between the HIV epidemic in SSA and the rise in NCDs, in particular cardiovascular disease (CVD) (4). This relationship is complex as the HIV infection itself, the treatment for HIV, and the timing of treatment during the disease course are all likely to affect the risk of CVD. While there is evidence that people living with HIV (PLHIV) have a higher risk of CVD than HIV-negative individuals (5), a possibly far more important driver of the epidemiological transition from acute infectious diseases to chronic NCDs in SSA is the massive scale-up of antiretroviral therapy (ART) (6). The survival of HIV-positive populations into old age due to ART will likely reveal CVD burdens previously “hidden” by high HIV mortality (6,7). In addition, there has been a growing concern that several antiretroviral drugs have side effects that increase patients’ risk of developing CVD. A systematic review found that PLHIV on ART had a higher risk of CVD than PLHIV not on ART, and the risk of developing CVD rose with increasing duration on ART (5). However, the effect of ART on CVD risk is likely to vary substantially between different antiretroviral drugs (5,8,9) and with the timing of ART initiation (as there is some evidence that earlier initiation of ART may decrease CVD risk (10)).

Weight gain following ART initiation is well documented (11–15). However, the weight gain on ART is likely to follow a prior weight loss. Weight loss, including severe wasting, is part of the natural history of HIV infection (16). This study focuses on the first 7 years of ART scale-up in SSA (between 2004 and 2010). Because treatment guidelines during this period recommended ART only for those whose HIV infection was relatively advanced, and because a large proportion of PLHIV enrolled in care long after their disease stage had advanced to the point of ART eligibility (17), many PLHIV were likely to have lost significant amounts of weight by the time they were initiated on ART. By greatly extending the life expectancy of these patients, ART is likely to have increased the proportion of individuals in the population who had lost weight due to HIV. It is unknown how rapidly and to what degree these ART patients regained the weight they had lost or indeed if their weight on ART eventually exceeded their weight at the time of infection.

A recent systematic review found that ART patients tended to have higher blood pressure (BP) and increased hypertension risk compared to HIV-positive patients not on ART (18). In addition, there is evidence that antiretroviral drugs directly interfere with the physiological mechanism of BP regulation by damaging the endothelial lining of blood vessels (19,20). Clinic- and laboratory-based studies, however, are unable to show how the scale-up of ART has affected BP and hypertension risk at the population level.

The distinct advantage of examining the relationship between ART and body mass index (BMI) in a population-based rather than clinic-based study is that it is possible to determine the population “net effect” of ART on weight. This net effect consists of two individual-level effects of ART: (1) a biological effect, the weight gain following ART initiation, and (2) a compositional effect, due to the increased survival of HIV-positive people in advanced disease stages, who are likely to have lost weight due to HIV infection. In addition, in a population-based study, it is possible to study trends in BMI (and BP) in the HIV-negative population, which provides insight into the population’s secular trends in cardiovascular risk independent of HIV infection and ART.

This study was carried out in a community with a very high prevalence of both HIV and obesity in rural KwaZulu-Natal, South Africa (6,21). Using data from two population-based anthropometric surveys—one conducted prior to the first HIV patients receiving ART in the area and the other when estimated ART coverage in the area had reached 25% (22)—we aimed to determine (1) the association between the ART scale-up and BMI and BP at the population level, and (2) the secular trend in BMI and BP in the HIV-negative population.

## Methods

### Study setting

**Figure 1:**
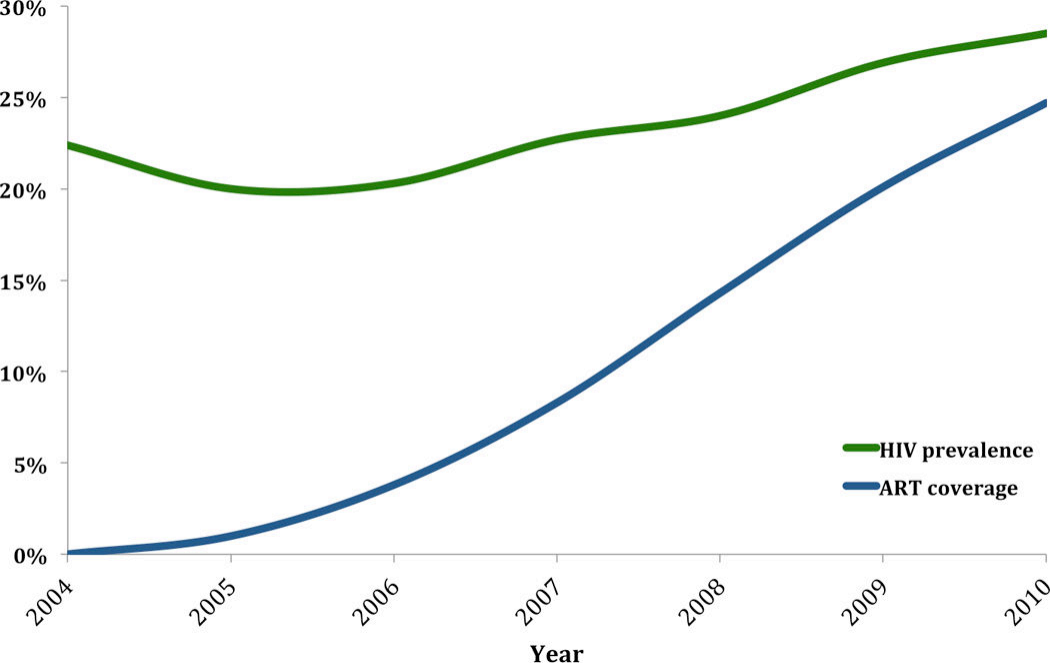
Human immunodeficiency virus (HIV) prevalence and antiretroviral therapy (ART) coverage among the 15- to 49-year-old population in the study community. ART coverage is the estimated percentage of people living with HIV aged 15 to 49 years who are on ART. [Color figure can be viewed at wileyonlinelibrary.com]

The two anthropometric surveys were conducted in the Africa Health Research Institute’s surveillance area, which is located in the largely rural Hlabisa subdistrict of uMkhanyakude district in KwaZulu-Natal, South Africa. The UMkhanyakude district is one of the poorest districts in South Africa (23). The surveillance population consists of an open cohort of more than 100,000 individuals. HIV prevalence in the surveillance area was 22% in 2004, the year of the first anthropometric survey, and 29% in 2010, when the second anthropometric survey was conducted (Figure 1) (22). The HIV surveillance which generated this HIV data is described in detail elsewhere (24).

### Study design

We conducted two cross-sectional anthropometric surveys, one before ART scale-up in 2004 and one in 2010 when an estimated 25% of HIV-positive individuals in the surveillance area were on ART (Figure 1) (22). In both surveys we measured weight, height, and BP using the WHO STEPS protocol (25). We selected a random sample of 30 subareas within the Africa Health Research Institute’s surveillance area for the anthropometric survey in 2004. For this analysis, the same 30 subareas were chosen for the data from the 2010 anthropometric survey. Eligibility criteria for participation in the surveys were (1) being a resident household member in one of the sampled subareas, and (2) being aged between 25 and 49 years for women and 25 and 54 years for men.

Data from the anthropometric surveys was matched with individuals’ HIV and ART status from the Africa Health Research Institute’s HIV surveillance data. The HIV surveillance is conducted through annual household visits, during which all adult household members are offered an HIV test. Within a 5-year period, about 80% of the individuals in this open cohort consent to HIV testing (26). If a household member consents to an HIV test, a small finger prick blood sample is taken. HIV status is then assessed by an enzyme-linked immunosorbent assay (ELISA) of EDTA anticoagulated blood samples in the Africa Health Research Institute’s virology laboratory, using a HIV-1/ HIV-2 ELISA assay (Vironostika; Organon Taknika, Boxtel, The Netherlands). In all HIV-positive samples, confirmatory HIV testing was carried out. None of the participants in either the 2004 or the 2010 survey had discordant HIV test results. Ethical approval for the anthropometric survey and HIV surveillance was granted by the Bio-Medical Ethics Committee of the University of KwaZulu-Natal.

### Definitions of overweight, obesity, and hypertension

We defined overweight or obesity as a BMI ≥25.0 kg/m^2^ and obesity as a BMI ≥30.0 kg/m^2^. Following the guidelines of the European Society of Cardiology (27), stage 1 hypertension was defined as a systolic BP (sBP) ≥140 mm Hg and/or a diastolic BP (dBP) ≥90 mm Hg, and stage 2 hypertension was defined as sBP ≥160 mm Hg and/or dBP ≥100 mm Hg.

### Statistical analysis

For both the 2004 and 2010 survey, we calculated the mean BMI, mean sBP, and mean dBP, as well as the prevalence of overweight and obesity and stage 1 and stage 2 hypertension. In sensitivity analyses we adjusted for changes in the age composition of the population between the two survey rounds. The statistical analysis was conducted using Stata 13.0 (State Corporation, College Station, TX).

## Results

For the 2004 survey, 3,000 individuals were contacted, of which 2,252 (75.1%) agreed to a height and weight measurement and 2,266 (75.5%) to a BP measurement. For the 2010 survey, 4,608 were contacted and 2,088 (45.3%) agreed to a height and weight measurement and 2,584 (55.3%) to BP measurement. Two hundred seventy-six individuals participated in both surveys.

### BMI and prevalence of overweight and obesity

Overweight and obesity prevalence is high among this population and was markedly higher among women than men in both 2004 and 2010 (Tables 1 and 2). Among women, mean BMI decreased from 29.9 kg/m^2^ in 2004 to 29.1 kg/m^2^ in 2010 (*P* = 0.002); among men, mean BMI decreased from 24.2 kg/m^2^ to 23.0 kg/m^2^ (*P*<0.001). Similarly, overweight and obesity prevalence decreased in both sexes with the decline being more marked for men.

When stratified by HIV status, we found that mean BMI decreased for women living with HIV from 28.2 to 27.1 kg/m^2^ (*P* = 0.026), but there was no statistically significant change in women who were HIV-negative and those with unknown HIV status. We also found no statistically significant differences in the proportion of women with overweight or obesity by HIV status except for a marginally significant (*P* = 0.061) decrease in the proportion of HIV-positive women with overweight or obesity from 63.5% (95% CI: 58.2–68.7%) to 57.1% (53.2–61.1%). For men, BMI did not change among men living with HIV but decreased among HIV-negative men from 24.2 to 23.0 kg/m^2^ (*P* = 0.004) and among those with unknown HIV status from 25.2 to 24.2 kg/m^2^ (*P* = 0.030). Both the proportion with overweight or obesity (BMI ≥25 kg/m^2^) and the proportion with obesity (BMI ≥30 kg/m^2^) decreased significantly among HIV-negative men from 32.4% to 23.8% and from 12.3% to 6.6%, respectively.

### BP and prevalence of hypertension

In the population, mean sBP decreased significantly in both women (from 123.0 to 118.2 mm Hg; *P* < 0.001) and men (from 128.4 to 123.2 mm Hg; *P* < 0.001). Mean dBP increased slightly in both sexes, from 79.6 to 81.4 mm Hg (*P* < 0.001) in women and from 79.0 to 80.7 mm Hg (*P* = 0.005) in men. When stratified by HIV status, mean sBP decreased between 2004 and 2010 among all three groups (HIV-positive, HIV-negative, and unknown HIV status) in both sexes. Mean dBP, on the other hand, increased slightly among HIV-negative women, women with an unknown HIV status, and HIV-negative men. There was no significant change in the proportion of adults with hypertension (stage 1 or 2 hypertension) or stage 2 hypertension in the aggregate population nor when stratified by sex and HIV status (Supporting Information Table S1).

The results presented in Tables 1 and 2 do not change substantially when adjusting for changes in the age composition of the population between the two survey rounds.

### Weight and BP by ART status

Stratifying the sample living with HIV in 2010 by ART status (Table 3), we find that mean BMI was higher among those not on ART (ART−) than those on ART (ART+) for both men and women. In addition, the proportion of ART− women with obesity was almost double that of ART+ women (31.6% vs. 16.2%; *P* < 0.001). Differences in sBP and dBP were only significant when adjusting for age with mean BP being higher among ART− than ART+. There were no significant differences in stage 1 and stage 2 hypertension between the ART− and ART+ populations.

**Figure 2:**
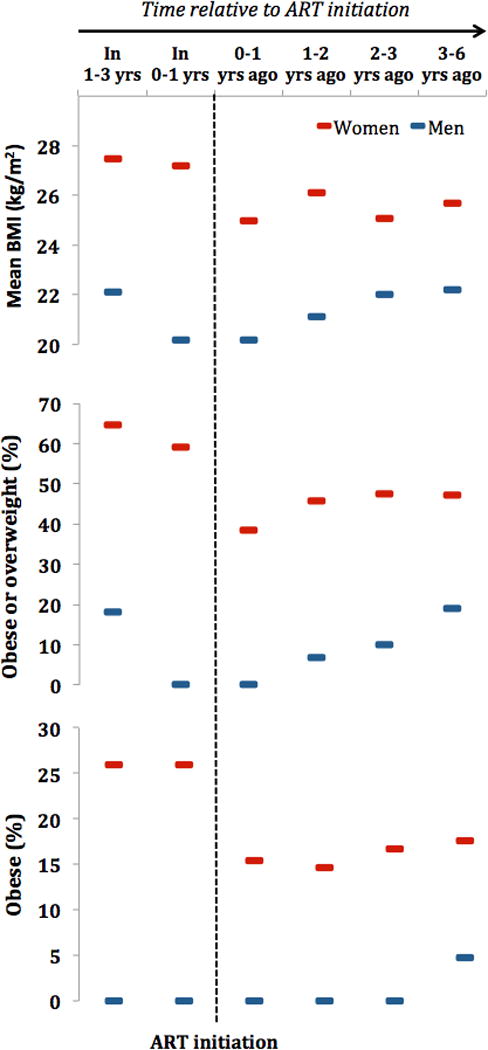
BMI, overweight, and obesity relative to the time of ART initiation by sex. ART, antiretroviral therapy; BMI, body mass index. The accompanying data, including 95% confidence intervals, can be found in Supporting Information Tables S1 and S2. [Color figure can be viewed at wileyonlinelibrary.com]

We further disaggregated this cross-sectional sample by time to ART initiation (for those ART− at the time of the 2010 survey) and time since ART initiation (for those ART+ at the time of the 2010 survey). Figure 2 (data with confidence intervals [CIs] shown in Supporting Information Table S1 and S2) depicts a general trend among both men and women: mean BMI, prevalence of BMI ≥25 kg/m^2^ (overweight or obesity), and prevalence of BMI ≥30 kg/ m^2^ (obesity) decreased up until shortly after ART initiation and then increased again with a higher duration on ART. Importantly, men who initiated ART within 1 to 3 years after the survey tended to fully recover their baseline BMI. In contrast, women tended not to fully recover their baseline BMI, even 3–6 years after ART initiation.

## Discussion

In an HIV-hyperendemic rural community in KwaZulu-Natal, South Africa, BMI decreased substantially in both sexes (by 0.8 BMI points among women and 1.2 BMI points among men) following the public-sector ART scale-up. Our results suggest that the population-level decline in BMI among women was likely caused by ART improving the survival of women living with HIV who had suffered substantial weight loss due to HIV disease. Among men, on the other hand, the population-level decrease in BMI seems largely due to the decline in BMI among those who were HIV-negative. These findings are plausible as the HIV treatment guidelines in place between 2004 and 2010 recommended starting ART at a CD4-cell count <200 cells/mm^3^ or when the patient is in WHO clinical stage IV (28). Thus, only the sickest HIV patients, and therefore those with the most significant HIV-related weight loss, were started on ART. Some of these patients died between the two survey rounds and were therefore not surveyed in 2010. In a recent study, we found that the ART scale-up in the study area has been accompanied by a greater reduction in HIV-related mortality among women than men (29). This observation implies that men with substantial HIV-related weight loss were more likely to have died (and therefore left the study population) despite the availability of ART than their female counterparts, thus increasing average BMI among HIV-positive men as compared to HIV-positive women.

Another possible reason for the decline in BMI among women but not men living with HIV is depicted in Figure 2. While this study is limited by the cross-sectional nature of the data, both men and women lose weight shortly before (and possibly for the first few months after) initiating ART. Once initiated on ART, however, those men who survive seem to regain their weight more quickly than their female peers. One possible explanation for this trend is food insecurity. In the same study area in rural KwaZulu-Natal, Patenaude et al. found that ART had a positive causal effect on the probability of any adult in the household missing a meal for financial reasons in the first 2 years after ART initiation (30). This effect returned to zero between 2 and 4 years after ART initiation, a trend that may be explained by the fact that the initial increase in food insecurity is due to the high immediate financial costs of attending ART care (31) (e.g., the cost of transport to the ART clinic and time lost from work), while the beneficial effects of ART on employment and income are delayed (32). It is plausible that men’s meals are prioritized over that of women in food insecure households (33), which may allow men to regain their weight more quickly after ART initiation compared with women.

Our 2010 BMI and BP findings for this poor rural community with a very high prevalence of HIV were similar to national estimates from the South African National Health and Nutrition Examination Survey carried out in 2012. However, while mean BMI, overweight, and obesity increased between 1998 and 2012 in South Africa (34,35), it decreased in the study area between our 2004 and 2010 surveys. Both nationally and in this community, BMI, overweight, and obesity were substantially higher among women than men in both 2004 and 2010. The reasons for this large difference by sex are still unclear and likely to be multifactorial (36). Importantly, however, while we found that mean BMI and the proportion of adults with overweight and obesity decreased between 2004 and 2010 for both sexes, the decline was more marked among men than women. The discrepancy in the prevalence of overweight and obesity by sex is therefore widening in this study population, which is also true for South Africa as a whole (34,35). The finding that BMI decreased in HIV-negative men from 24.2 to 23.0 kg/m^2^ (*P* = 0.004) while there was no significant change among HIV-negative women suggests that this divergence might be occurring independently of HIV status. However, firm conclusions are limited by the possibility of a relationship between BMI and the probability of acquiring HIV.

We found that BP and hypertension prevalence were lower among PLHIV than those not infected with HIV (Tables 1 and 2, Supporting Information Table S1). This could be a reflection of a higher BP among individuals with an increased risk of acquiring HIV. An alternative explanation is that HIV-related weight loss led to a reduction in BP. In addition, we found that mean BP tended to be higher among PLHIV with a longer duration on ART (Supporting Information Tables S2 and S3). This may be explained by weight gain on ART-induced damage to the endothelial lining (19,20). However, unlike a recent systematic review, which found a positive association between ART and BP (18), we found lower sBP and dBP (after adjusting for age) among those on ART than PLHIV who were not on ART.

This study has several limitations. First, refusal to participate in the survey was comparatively high, which may have compromised the representativeness of our sample. More specifically, if those who declined to participate in the survey had a different BMI or BP than those who participated, our results for both the 2004 and 2010 survey a would fail to be representative of the entire study population at each time point. Furthermore, the refusal rate was higher in 2010 as compared with 2004. Thus, if the additional proportion of the population who declined in 2010 as compared with 2004 had a different BMI and BP than those who participated in 2004, the changes in BMI and BP between the two time points might be biased. Second, the implications of this study for health policy would have been strengthened if biomarkers of cardiovascular risk had been assessed, such as biomarkers for diabetes and a lipid profile. However, such data was not available at the time of this analysis. Third, additional anthropometric data would have allowed further insight into the relationship between ART and CVD at the population level. However, a systematic review found that two important anthropometric measures, waist circumference and waist-to-hip ratio, were not stronger predictors of CVD than BMI (37). Fourth, this study can only ascertain the association between the ART scale-up and BMI and BP under the 2004 South African HIV treatment guidelines, which recommend the initiation of ART for PLHIV in advanced disease stages (28). The association between ART scale-up and BMI and BP may be substantially different when PLHIV in earlier disease stages are eligible to receive ART.

## Conclusion

There have been fears that the ART scale-up contributes to the rise in CVD in SSA by increasing the prevalence of overweight and obesity (6). We found that mean BMI, sBP, and the proportion of the population with overweight and obesity decreased in the first years of the public-sector ART scale-up in a rural community in KwaZulu-Natal with high overall levels of both adiposity and HIV. Overweight and obesity prevalence was lower around the time of ART initiation and higher among those with a longer duration on ART, similar to findings by Feigl et al. (38). Overall, our findings suggest that the compositional effect of ART—increasing the survival of HIV-positive populations with large average weight loss—outweighs the biological ART effect—increasing BMI following ART initiation. Where overall levels of adiposity are high, as in the community in which this study took place, the population net effect of the ART scale-up on BMI provides an opportunity to intervene to ensure that HIV-positive people on ART maintain healthy weight levels in the long run.

## Supplementary Material

supplementary

## Figures and Tables

**TABLE 1:** BMI and blood pressure (BP) among women

	Entire female population			HIV-positive female population			HIV-negative female population			Female population with unknown HIV status		
	2004	2010	*P*	2004	2010	*P*	2004	2010	*P*	2004	2010	*P*
***n***	1,505	1,491		323	609		564	646		611	236	
**Mean BMI (kg/m**^2^**)**	29.9 (29.6–30.3)	29.1 (28.8–29.5)	0.002	28.2 (27.3–29.1)	27.1 (26.6–27.6)	0.026	30.9 (30.3–31.5)	30.9 (30.3–31.4)	0.998	29.9 (29.4–30.5)	29.5.(28.7–30.4)	0.444
**Overweight**^a^ **or obesity**^b^ **(%)**	72.9 (70.6–75.1)	68.8 (66.5–71.2)	0.014	63.5 (58.2–68.7)	57.1 (53.2–61.1)	0.061	76.8 (73.3–80.3)	78.6 (75.5–81.8)	0.437	74.3 (70.8–77.8)	72.0 (66.3–77.8)	0.501
**Obesity**^b^ **(%)**	42.8 (40.3–45.3)	40.0 (37.6–42.5)	0.127	27.9 (23.0–32.8)	26.3 (22.8–29.8)	0.602	51.2 (47.1–55.4)	51.2 (47.4–55.1)	0.999	42.7 (38.8–46.6)	44.9 (38.6–51.3)	0.563
***n***	1,557	1,825		335	706		580	778		634	341	
**Mean systolic BP (mm Hg)**	123.0 (122.0–123.9)	118.2 (117.4–119.0)	<0.001	120.8 (118.9–122.7)	115.1 (113.9–116.3)	<0.001	125.1 (123.6–126.7)	120.4 (119.1–121.6)	<0.001	122.1 (120.6–123.6)	119.7 (117.5–122.0)	0.068
**Mean diastolic BP (mm Hg)**	79.6 (79.0–80.2)	81.4 (80.8–82.0)	<0.001	79.0 (77.8–80.2)	79.6 (78.7–80.5)	0.401	80.3 (79.3–81.4)	82.8 (81.9–83.7)	<0.001	79.2 (78.2–80.2)	82.1 (80.6–83.6)	0.001

95% confidence intervals are shown in parentheses. Means were compared using student’s *t*-test and proportions using a *z*-test.

a Overweight was defined as a BMI ≥25 kg/m^2^ and <30 kg/m^2^.

b Obesity was defined as a BMI ≥30 kg/m^2^.

BMI, body mass index; HIV, human immunodeficiency virus.

**TABLE 2:** BMI and blood pressure (BP) among men

	Entire male population			HIV-positive male population			HIV-negative male population			Male population with unknown HIV status		
	2004	2010	*P*	2004	2010	*P*	2004	2010	*P*	2004	2010	*P*
***n***	747	597		156	194		324	290		263	113	
**Mean BMI (kg/m**^2^)	24.2 (23.8–24.7)	23.0 (22.6–23.3)	<0.001	22.5 (22.0–23.0)	22.5 (22.0–23.0)	0.420	24.2 (23.5–24.8)	23.0 (22.5–23.5)	0.004	25.2 (24.5–26.0)	24.2 (23.1–25.4)	0.030
**Overweight**^a^ **or obesity (%)**	31.9 (28.5–35.2)	22.3 (18.9–25.6)	<0.001	19.9 (13.6–26.1)	16.5 (11.3–21.7)	0.414	32.4 (27.3–37.5)	23.8 (18.9–28.7)	0.018	38.4 (32.5–44.3)	28.3 (20.0–36.6)	0.061
**Obesity**^b^ **(%)**	11.7 (9.4–14.0)	7.0 (5.0–9.1)	0.004	4.5 (1.2–7.7)	4.6 (1.7–7.6)	0.946	12.3 (8.8–15.9)	6.6 (3.7–9.4)	0.015	14.8 (10.5–19.1)	12.4 (6.3–18.5)	0.533
***n***	761	759		157	239		332	336		268	184	
**Mean systolic BP (mm Hg)**	128.4 (127.1–129.7)	123.2 (122.1–124.4)	<0.001	124.8 (122.2–127.5)	120.3 (118.6–122.1)	0.004	129.3 (127.4–131.3)	125.7 (123.9–127.6)	<0.001	129.5 (127.3–131.7)	122.4 (119.8–125.0)	<0.001
**Mean diastolic BP (mm Hg)**	79.0 (78.1–79.8)	80.7 (79.9–81.6)	0.005	77.6 (75.7–79.5)	78.7 (77.3–80.1)	0.346	78.3 (77.0–79.7)	81.9 (80.6–83.3)	<0.001	80.7 (79.3–82.2)	81.2 (79.5–83.0)	0.662

95% confidence intervals are shown in parentheses. Means were compared using student’s *t*-test and proportions using a *z*-test.

a Overweight was defined as a BMI ≥25 kg/m^2^ and <30 kg/m^2^.

b Obesity was defined as a BMI ≥30 kg/m^2^.

BMI, body mass index; HIV, human immunodeficiency virus.

**TABLE 3:** BMI and blood pressure (BP) in 2010 among people living with HIV, by ART status

	Female			Male		
	ART−	ART+	*P*	ART−	ART+	*P*
***n***	399	210		132	62	
**Mean BMI (kg/m**^2^**)**	28.0 (27.4–28.6)	25.5 (24.7–26.3)	<0.001	22.5 (21.7–23.3)	21.4 (20.5–22.2)	0.074
**Overweight or obesity (%)**^a^	63.7 (58.9–68.4)	44.8 (38.0–51.5)	<0.001	19.7 (12.9–26.5)	9.7 (2.3–17.0)	0.080
**Obesity (%)**^b^	31.6 (27.0–36.1)	16.2 (11.2–21.2)	<0.001	6.1 (2.0–10.1)	1.6 (0.0–4.7)	0.170
***n***	450	256		158	81	
**Not adjusted for age**						
**Mean systolic BP (mm Hg)**	115.2 (113.6–116.8)	114.9 (112.9–116.9)	0.796	121.4 (119.1–123.6)	118.3 (115.5–121.2)	0.107
**Mean diastolic BP (mm Hg)**	80.0 (78.9–81.1)	78.8 (77.4–80.2)	0.208	79.6 (77.8–81.3)	77.0 (74.8–79.1)	0.077
**Adjusted for age**						
**Mean systolic BP (mm Hg)**	115.9 (114.4–117.3)	113.7 (111.8–115.7)	0.086	121.90 (119.7–124.1)	117.3 (114.2–120.4)	0.020
**Mean diastolic BP (mm Hg)**	80.4 (79.4–81.5)	78.1 (76.7–79.5)	0.010	80.0 (78.3–81.7)	76.1 (73.7–78.5)	0.010

95% confidence intervals are shown in parentheses. Means were compared with the student’s *t*-test and proportions using a *z*-test. None of the BMI comparisons changed significantly when adjusting for age. The numbers shown here are not adjusted for age.

a Defined as BMI ≥25 kg/m^2^.

b Defined as a BMI ≥30 kg/m^2^.

ART−, not on antiretroviral therapy at the time of the survey; ART+, on antiretroviral therapy at the time of the survey; BMI, body mass index.
