# Supplementary material for: Population-Level Decline in BMI and Systolic Blood Pressure Following Mass HIV Treatment: Evidence from Rural KwaZulu-Natal
Source: Obesity (Silver Spring). Author manuscript; Available in PMC 2018 May 9. (PMC5940485; doi:10.1002/oby.21663)
Supplement: supplementary [file NIHMS870775-supplement-supplementary.pdf]

Appendix 1. Prevalence of hypertension in the study population

	Population			HIV-infected			HIV-uninfected			Unknown HIV-status[1]		
	**2004**	**2010**	**p-value**	**2004**	**2010**	**p-value**	**2004**	**2010**	**p-value**	**2004**	**2010**	**p-value**
Women	1,557	1,825		335	706		580	778		634	341	
**Hypertension (%)[2]**	24.1 (22.0 – 26.3)	24.1 (22.1 – 26.1)	0.979	21.1 (16.8 – 25.5)	19.3 (16.4 – 22.2)	0.480	26.2 (22.6 – 29.8)	27.6 (24.5 – 30.8)	0.558	24.0 (20.7 – 27.3)	26.1 (21.4 – 30.8)	0.472
**Stage 2 hypertension (%)[3]**	8.3 (7.0 – 9.7)	9.5 (8.2 – 10.9)	0.230	7.1 (4.4 – 9.9)	7.1 (5.2 – 9.0)	0.972	10.3 (7.9 – 12.8)	11.6 (9.3 – 13.8)	0.477	7.3 (5.2 – 9.3)	10.0 (6.8 – 13.2)	0.143
Men	761	759		157	239		332	336		268	184	
**Hypertension (%)[2]**	26.8 (23.7 – 30.0)	25.0 (22.0 – 28.1)	0.430	22.9 (16.4 – 29.5)	19.2 (14.2 – 24.2)	0.376	27.7 (22.9 – 32.5)	26.8 (22.1 – 31.5)	0.788	28.4 (23.0 – 33.8)	29.3 (22.8 – 35.9)	0.819
**Stage 2 hypertension (%)[3]**	8.8 (6.8 – 10.8)	7.5 (5.6 – 9.4)	0.357	5.7 (2.1 – 9.4)	5.4 (2.6 – 8.3)	0.901	8.7 (5.7 – 11.8)	9.2 (6.1 – 12.3)	0.824	10.8 (7.1 – 14.5)	7.1 (3.4 – 10.8)	0.177

95% confidence intervals are shown in brackets. Means were compared with the student's t-test and proportions using a z-test.

[1] HIV status was recorded as unknown if a participant could not be linked to the Africa Centre for Population Health's HIV surveillance data, most commonly because he/she did not consent to HIV testing during past surveillance rounds.

[2] Hypertension was defined as systolic BP ≥ 140mmHg and/or diastolic BP ≥ 90mmHg.

[3] Stage 2 hypertension was defined as systolic BP ≥ 160mmHg and/or diastolic BP ≥ 100mmHg.

Appendix 2. BMI and blood pressure in 2010 among women living with HIV, by length on ART[1]

	**Initiated on ART after the survey**		**Initiated on ART before the survey**			
	In 1-3 years	***In 0-1 year***	***0-1 year ago***	***1-2 years ago***	***2-3 years ago***	***3-6 years ago***
n	54	54	52	48	42	68
**Mean BMI (kg/m^2)**	27.5 (25.9 – 29.0)	27.2 (25.5 – 28.8)	25.0 (23.6 – 26.4)	26.1 (24.0 – 28.2)	25.1 (23.6 – 26.6)	25.7 (24.3-27.1)
**Overweight or obese[2] (%)**	64.8 (52.1 – 77.6)	59.3 (46.2 – 72.4)	38.5 (25.2 – 51.7)	45.8 (31.7 – 59.9)	47.6 (32.5 – 62.7)	47.1 (35.2 – 58.9)
**Obese[3] (%)**	25.9 (14.2 – 37.6)	25.9 (14.2 – 37.6)	15.4 (5.6 – 25.2)	14.6 (4.6 – 24.6)	16.7 (5.4 – 27.9)	17.6 (8.6 – 26.7)
n	61	63	60	59	52	85
**Mean systolic BP (mmHg)**	117.5 (113.3 – 121.6)	113.0 (108.6 – 117.5)	112.1 (108.4 – 115.8)	115.5 (111.3 – 119.6)	115.3 (111.2 – 119.5)	116.1 (112.1 – 120.1)
**Mean diastolic BP (mmHg)**	81.6 (78.5 – 84.7)	78.0 (74.8 – 81.1)	77.6 (74.9 – 80.3)	78.3 (75.5 – 81.2)	79.6 (76.8 – 82.4)	79.5 (76.7 – 82.4)
**Hypertension[4] (%)**	23.0 (12.4 – 33.5)	17.5 (8.1 – 26.8)	13.3 (4.7 – 21.9)	15.3 (6.1 – 24.4)	19.2 (8.5 – 29.9)	21.2 (12.5 – 29.9)
**Stage 2 hypertension[5] (%)**	9.8 (2.4 – 17.3)	7.9 (1.3 – 14.6)	5.0 (0.0 – 10.5)	5.1 (0.0 – 10.7)	5.8 (0.0 – 12.1)	5.9 (0.9 – 10.9)

Abbreviations: BMI = Body Mass Index; ART = antiretroviral therapy; BP = blood pressure

[1] 95% confidence intervals are provided in brackets after the point estimate.

[2] Defined as a BMI ≥25 kg/m^2.

[3] Obesity was defined as a BMI ≥30 kg/m^2.

[4] Hypertension was defined as systolic BP ≥140 mmHg or diastolic BP ≥90 mmHg.

[5] Stage 2 hypertension was defined as systolic BP ≥ 160mmHg and/or diastolic BP ≥ 100mmHg.

Formatted Table

Formatted Table

Appendix 3. BMI and blood pressure in 2010 among men living with HIV, by length on ART

	**Initiated on ART after the survey**		**Initiated on ART before the survey**			
	In 1-3 years	***In 0-1 year***	***0-1 year ago***	***1-2 years ago***	***2-3 years ago***	***3-6 years ago***
n	11	13	16	15	10	21
**Mean BMI (kg/m^2)**	22.1 (19.7 – 24.5)	20.2 (19.0 – 21.5)	20.2 (19.1 – 21.2)	21.1 (19.3 – 22.8)	22.0 (20.4 – 23.7)	22.2 (20.2 – 24.2)
**Overweight or obese[2] (%)**	18.2 (0.0 – 41.0)	0.0 (0.0 – 0.0)	0.0 (0.0 – 0.0)	6.7 (0.0 – 19.3)	10.0 (0.0 – 28.6)	19.0 (2.3 – 35.8)
**Obese[3] (%)**	0.0 (0.0 – 0.0)	0.0 (0.0 – 0.0)	0.0 (0.0 – 0.0)	0.0 (0.0 – 0.0)	0.0 (0.0 – 0.0)	4.8 (0.0 – 13.9)
n	13	15	25	16	14	26
**Mean systolic BP (mmHg)**	120.3 (110.9 – 129.8)	113.5 (106.5 – 120.5)	113.8 (108.5 – 119.0)	114.8 (107.2 – 122.3)	123.6 (116.6 – 130.7)	122.0 (117.6 – 126.5)
**Mean diastolic BP (mmHg)**	82.8 (76.6 – 89.0)	74.1 (69.0 – 79.3)	75.4 (70.9 – 79.9)	74.0 (68.9 – 79.1)	78.6 (73.8 – 83.3)	79.4 (75.7 – 83.1)
**Hypertension[4] (%)**	30.8 (5.7 – 55.9)	13.3 (0.0 – 30.5)	12.0 (0.0 – 24.7)	6.3 (0.0 – 18.1)	21.4 (0.0 – 42.9)	26.9 (9.9 – 44.0)
**Stage 2 hypertension[5] (%)**	7.7 (0.0 – 22.2)	0.0 (0.0 – 0.0)	4.0 (0.0 – 11.7)	6.3 (0.0 – 18.1)	0.0 (0.0 – 0.0)	0.0 (0.0 – 0.0)

Abbreviations: BMI = Body Mass Index; ART = antiretroviral therapy; BP = blood pressure

[1] 95% confidence intervals are provided in brackets after the point estimate.

[2] defined as a BMI ≥25 kg/m^2.

[3] Obesity was defined as a BMI ≥30 kg/m^2.

[4] Hypertension was defined as systolic BP ≥140 mmHg or diastolic BP ≥90 mmHg.
